# Complete Genome Sequence of Classical Swine Fever Virus Subgenogroup 2.1 from Assam, India

**DOI:** 10.1128/genomeA.01437-14

**Published:** 2015-01-22

**Authors:** Anuj Ahuja, Uttaran Bhattacharjee, Amit Kumar Chakraborty, Amarjit Karam, Sandeep Ghatak, Kekungu Puro, Samir Das, Ingudam Shakuntala, Nidhi Srivastava, S. V. Ngachan, Arnab Sen

**Affiliations:** a Division of Animal Health, ICAR RC for NEH Region, Umiam, Meghalaya, India; b Department of Bioscience and Biotechnology, Banasthali University, Rajasthan, India

## Abstract

We report the complete genome sequence of a classical swine fever virus (genogroup 2.1), isolated from an outbreak in Assam, India. This particular isolate showed a high degree of genetic variation within the subgenogroup 2.1 and may serve as a potential reference strain of the 2.1 genogroup of classical swine fever virus (CSFV) in the Indian subcontinent.

## GENOME ANNOUNCEMENT

Classical swine fever (CSF) is a World Organization for Animal Health (OIE) notifiable, economically important, highly contagious viral disease of domestic pigs and wild boars caused by classical swine fever virus (CSFV). CSFV belongs to the *Pestivirus* genus within the family Flaviviridae. CSFV is genetically and antigenically related to other pestiviruses, including bovine viral diarrhea virus (BVDV) and border disease virus (BDV). CSFV possesses an enveloped, single-stranded, nonsegmented positive-sense RNA genome of approximately 12.3 kb, with a single open reading frame (ORF) which co- and posttranslationally processes into 11 polypeptides, flanking with 5′ and 3′ nontranslated regions (NTRs). CSFV has one serotype divided into three major genogroups, 1, 2, and 3, each comprising three to four subgenogroups (1, 2).

CSF is endemic in India (3). Pig rearing is an important agricultural activity in the northeastern states of India, which have the highest pig population. Several outbreaks of CSF have been reported from the region (4–6). Due to a long and porous international boundary, this region faces threats of transboundary transmission of exotic CSFV strains. Recent studies have shown that subgenogroup 1.1 is prevalent in India (7), with few reports of genogroup 2 (5, 6, 8).

Total RNA was extracted from blood samples by using a QIAamp viral RNA minikit (Qiagen). Extracted RNA was reverse transcribed using a RevertAid first-strand cDNA synthesis kit (Thermo Scientific) employing a random hexamer primer or specific primers designed against CSFV genome sequences. The whole genome was amplified by using overlapping fragments and cloned into a pTZ57R/T vector and sequenced using T7 and M13 primers or PCR product sequenced directly on an ABI Prism 3500xL DNA sequencer. Sequenced fragments were aligned and assembled to generate the whole-genome sequence of 12,289 nucleotides (nt). The polyprotein-coding sequence was 11,697 nt long and encoded 3,898 amino acids.

The Assam-G4 isolate genome sequence showed 93% homology with the Paderborn strain (accession no. GQ902941) at the nucleotide level and 96.4% at the amino acid level. On comparison with the Paderborn strain, the Assam-G4 isolate revealed sequence homology of 92% with the 5′ NTR, 93% with the Npro gene, 93% with the C gene, 93% with the Erns gene, 93% with the E1 gene, 92% with the E2 gene, 94% with the P7 gene, 92% with the NS2 gene, 93% with the NS3 gene, 96% with the NS4A gene, 93% with the NS4B gene, 91% with the NS5A gene, 92% with the NS5B gene, and 92% with the 3′ NTR.

Since the full-length E2-encoding region (1119 nt) is considered most suitable for phylogenetic analysis of CSFV (2), a phylogenetic tree was constructed by taking the E2 region from 48 previously reported sequences. Results indicated that the Assam-G4 isolate phylogenetically clustered in the subgenogroup 2.1, although on comparison a separate clade was evident with regard to the whole E-2 gene sequence (unpublished data, 2014).

This is the first report of a complete genome sequence of subgenogroup 2.1 CSFV from India. This genome sequence data may serve as a reference for the subgenogroup 2.1 isolates from the Indian subcontinent. More genome sequence information for subgenogroup 2.1 isolates would help in understanding of the divergence in this emerging genogroup of CSFV in India.

### Nucleotide sequence accession number.

The complete genome sequence of CSFV Assam-G4 has been deposited in GenBank under accession no. KM362426.
